# Supplementary material for: Splice-Junction-Based Mapping of Alternative Isoforms in the Human Proteome
Source: Cell Rep. Author manuscript; Available in PMC 2020 Jan 15. (PMC6961840; doi:10.1016/j.celrep.2019.11.026)

A

sp|Q96T60|PNKP_HUMAN|ENSG00000039650|RI1|118|chr19|49864236|49864403|−0|r37|T1
GVSEAWSPPLAPGHPCR q value: 0.002545 Tr_novel:TRUE RefSeq_Novel:TRUE
Search result spec prec mz: 606.9651 Actual spec prec mz: 606.96506
Fragments matched per AA: 2.24 Proportion of top 20 peaks matched: 0.15

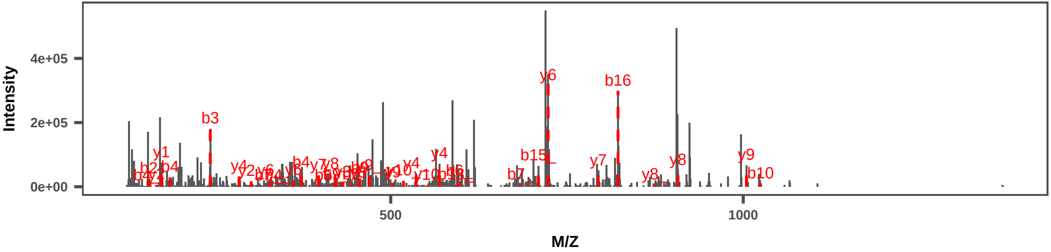

B

Scatterplot of predicted elution time
Fitting R2: 0.89
Novel peptide residual Z score: −0.877
Number of peptides: 971

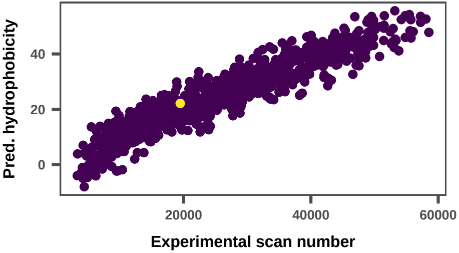

C

Distributions of residuals from best−fit line
of predicted RT vs Expt. scan number
Line: Z score of novel peptide
Z: −0.877

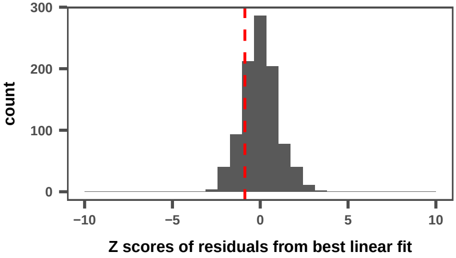

Supplement: 2 [file NIHMS1546469-supplement-2.zip › DF1/PXD010154/Pancreas/Pancreas_6_PNKP_GVSEAWSPPLAPGHPCR.pdf]
